# Imaging drugs, metabolites and biomarkers in rodent lung: a DESI MS strategy for the evaluation of drug-induced lipidosis

**DOI:** 10.1007/s00216-019-02151-z

**Published:** 2019-11-27

**Authors:** Alex Dexter, Rory T. Steven, Aateka Patel, Lea Ann Dailey, Adam J. Taylor, Doug Ball, Jan Klapwijk, Ben Forbes, Clive P. Page, Josephine Bunch

**Affiliations:** 1 National Physical Laboratory, Teddington, London, TW11 0LW UK; 2 Institute of Pharmaceutical Science, King’s College London, London, WC2R 2LS UK; 3 Martin-Luther-Universität Halle-Wittenberg, 06108 Halle, Saxony-Anhalt Germany; 4 Immunoinflammation TAU, GlaxoSmithKline, Stevenage, SG1 2NY UK; 5 Department of Surgery and Cancer, Faculty of Medicine, Imperial College, London, SW7 1LY UK

**Keywords:** Imaging mass spectrometry, BMP, Pharmaceuticals, Amiodarone, Mass spectrometry imaging

## Abstract

Within drug development and pre-clinical trials, a common, significant and poorly understood event is the development of drug-induced lipidosis in tissues and cells. In this manuscript, we describe a mass spectrometry imaging strategy, involving repeated analysis of tissue sections by DESI MS, in positive and negative polarities, using MS and MS/MS modes. We present results of the detected distributions of the administered drug, drug metabolites, lipid molecules and a putative marker of lipidosis, di-docosahexaenoyl (22:6)-bis(monoacylglycerol) phosphate (di-22:6-BMP). A range of strategies have previously been reported for detection, isolation and identification of this compound, which is an isomer of di-docosahexaenoic (22:6 n-3) phosphatidylglycerol (di-22:6 PG), a commonly found lipid that acts as a surfactant in lung tissues. We show that MS imaging using MS/MS can be used to differentiate these compounds of identical mass, based upon the different distributions of abundant fragment ions. Registration of images of these fragments, and detected drugs and metabolites, is presented as a new method for studying drug-induced lipidosis in tissues.

**Electronic supplementary material:**

The online version of this article (10.1007/s00216-019-02151-z) contains supplementary material, which is available to authorized users.

**Figure:**
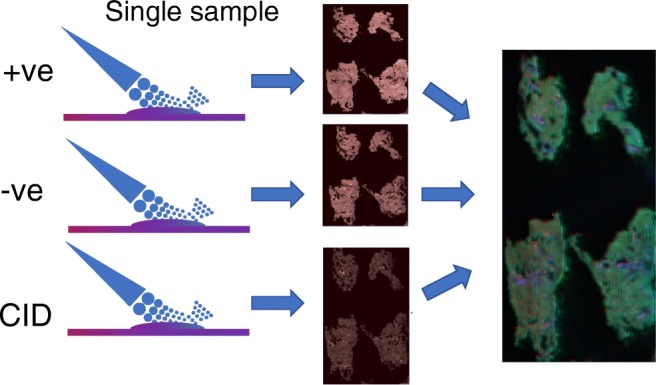
Graphical abstract

## Introduction

Mass spectrometry imaging (MSI) maps the spatial distribution of hundreds to thousands of molecules from a sample. It is frequently used in drug pharmacokinetics and toxicology to study the spatial distribution of drugs and metabolites in thin tissue sections [1, 2]. In addition to being able to measure drugs and exogenous metabolites in tissue, MSI can also be used to image endogenous metabolites, proteins or lipids within a tissue [3, 4]. Within drug development and pre-clinical trials, a common, significant and poorly understood barrier to success is the development of lipidosis in tissues and cells as a result of administered treatments. A deeper understanding of lipid changes occurring as a result of drug dosing will allow better selection of compounds for pre-clinical trials, and thus decrease the number of animals required. Amiodarone is a good model drug compound to study these effects, as it and its metabolite, desethylamiodarone, are readily detected by MSI in drug-treated mice [5] and oral dosing of amiodarone has been shown to induce pulmonary lipidosis, along with other adverse toxicological effects [6]. One previously reported putative marker of lipidosis is an increase in di-docosahexaenoyl (22:6)-bis(monoacylglycerol) phosphate (di-22:6-BMP), a lipid species associated with drug-induced lipidosis found within lysosome and endosomes [7]. Other amiodarone-induced lipid alterations have also been studied by MALDI MSI by Kashimura et al. primarily focused on changes in phosphocholine lipids [8]. They showed that in addition to di-22:6-BMP, amiodarone also increases levels of phosphocholine lipids in lung, spleen and lymph node tissues. Additionally, the metabolic pathway of amiodarone has been well-studied and characterised [9]. The lipidosis biomarker di-22:6-BMP has also been associated with other adverse effects, such as cerebral ischaemia which has been observed by DESI and MALDI MSI in negative ion mode [10]. One of the significant challenges associated with analysing di-22:6-BMP is that it is isomeric with di-docosahexaenoic (22:6 n-3) phosphatidylglycerol (di-22:6 PG), a commonly found lipid that acts as a surfactant in lung tissues [11]. Previously, separation and identification of di-22:6-BMP and di-22:6 PG have required either HPLC or ion mobility–based separations [12, 13].

In order to measure the spatial distribution of all these molecules, multiple polarities would be needed. Further, in order to confirm both their identity and spatial location, a combination of mass spectrometry (MS) and tandem MS modalities is required. This could be achieved by analysing multiple tissue sections, but this then presents additional registration challenges, as tissues will show changes in structures between sections, particularly in areas such as tumour microenvironments, or in lung tissues, which are prone to tears and deformation in the sectioning process. After analysis by MSI, there is often still a significant portion of tissue remaining; therefore, a much better route to gain the information needed would be by repeat analysis of the same tissue section. Repeat analysis of a single section by MSI was used by Eberlin et al. to analyse proteins and lipids from a single tissue section [14]. In their methods, DESI was first used to acquire images of lipids, followed by MALDI for protein analysis. More recently, Steven et al. performed repeat analysis of a single section by MALDI MSI to acquire data from a single tissue using multiple matrices and to perform MS and multiple MS/MS analyses [15].

Another means to obtain more information from a sample by MSI is to perform analyses in both positive and negative polarities. Thomas et al. showed that using MALDI matrices that readily ionise molecules in both positive and negative modes offers coverage of lipids that cannot be achieved by just one polarity [16]. By undersampling with the laser, they were able to acquire images in both polarities from a single section, but at a compromise of spatial resolution. This has recently been demonstrated at 25 μm [17] and 10 μm [18] pixel size; however, this is not possible for DESI MSI due to the much larger sampling probe size. True dual polarity analysis can be carried out, but requires custom instrumentation using two mass analysers [19].

This paper presents multiple repeat analyses of a single tissue section by DESI MSI using different polarities and modalities. These are used to measure the spatial distribution and confirm the identities of amiodarone, as well as endogenous and exogenous metabolites from a single tissue section.

## Methods

### Chemicals

Methanol (LC-MS grade) was purchased from Fisher Scientific (Leicestershire, UK); purified water from was purchased from ELGA Purelab Option (Marlow, UK); and raffinose pentahydrate (≥ 98%) and amiodarone hydrochloride (≥ 98%) were purchased from Sigma-Aldrich (Dorset, UK) and used as supplied. Superfrost Plus (Thermo Scientific, Waltham, MA, USA) glass slides were used for all experiments.

### Animal dosing

Male Wistar Han rats were purchased from Charles River (Wilmington, MA, USA) at 10–11 weeks old and approximately 300 g. Inhalation dosing of amiodarone aerosol was carried out using a capsule-based aerosol generator (CBAG) mechanism which is a dry powder inhaler (DPI) dosing system [20]. Dosing was carried out as follows: on days − 32, a target dose of 10 mg/kg amiodarone was delivered, and on days − 2, − 1 and 0, target doses of 30, 10 and 10 mg/kg amiodarone were delivered over a 30-min period. No adverse effects were observed with this dose of amiodarone. Animals were sacrificed on days 1 and 7 according to Animals (Scientific Procedures) Act 1986 (UK) and European Directive 2010/63/EU. Control animals were dosed by same method with air only. Six control and six drug-dosed animals were used within this study.

### Sample preparation

Lungs from dosed and control animals were excised, dissected into individual lobes and flash frozen in liquid nitrogen. Sample transport was subsequently carried out on dry ice. Samples were sectioned at 14 μm using a cryo-microtome (CM 1850, Leica, Milton Keynes, UK), thaw-mounted onto glass microscope slides (Superfrost Plus, Thermo Fisher, Waltham, MA, USA) and stored at − 80 °C until needed. Prior to use, sectioned samples were transferred immediately from − 80 °C storage into a vacuum desiccator and left for ~ 30 min to warm to room temperature and dry the tissue, thus minimising condensation on the sample and reducing any further metabolic activity. For analysis by DESI, samples were used with no additional preparation. Three sets of sections (three slides) were used in total: (A) 16 sections were sampled via the repeat analysis protocol to provide positive and negative mode data, (B) 2 sections were sampled in positive mode MS followed by MS/MS, and (C) 2 sections were sampled in negative mode MS and MS/MS.

### Mass spectrometry imaging

**Fig. 1:**
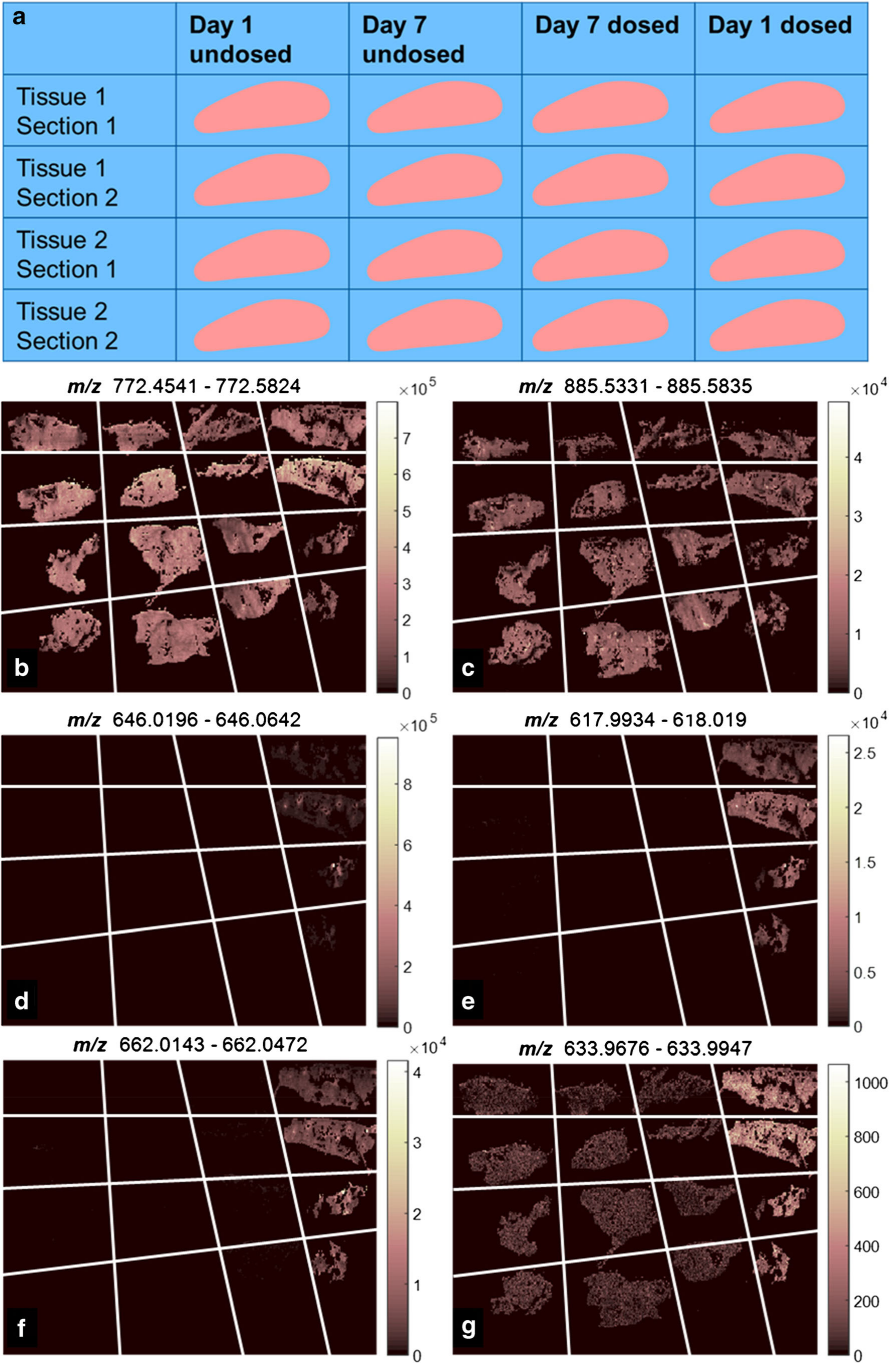
Reference key for the location of the different lung treatments (**a**), and selected ion images for example positive and negative mode lipids (**b** and **c** tentatively assigned by mass as PC 32:0 [M+K]^+^ and PI 38:4 [M−H]^−^ from literature [22, 23]). Along with amiodarone (**d**), N-desyl amiodarone (**e**) and metabolites M11 (**f**) and M8 (**g**), metabolite M8 has an isobaric species present which is found in all tissue samples. Full image area for these data is 46.5 × 23.3 mm

**Fig. 2:**
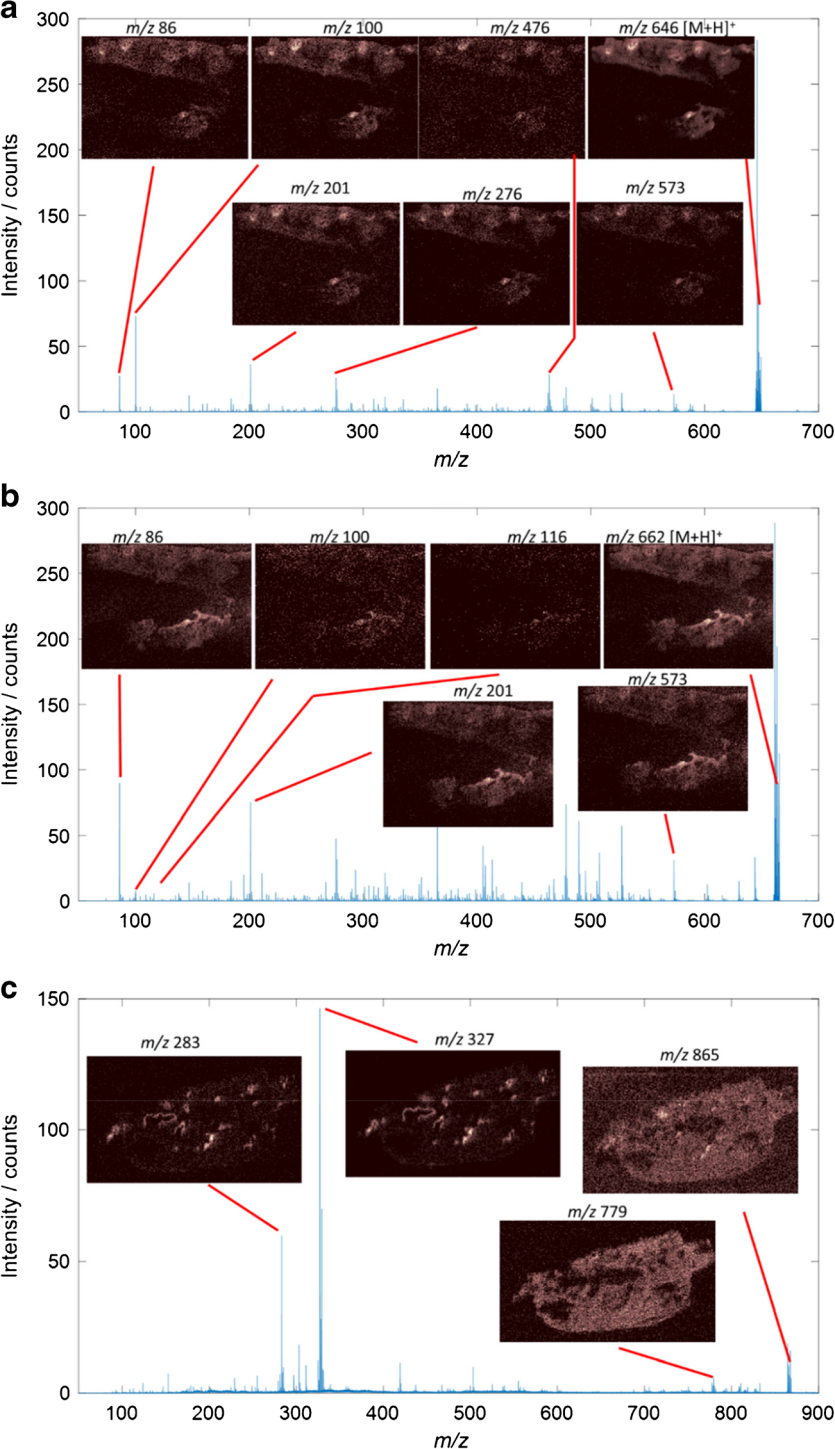
Mean MS/MS spectra from selected regions of interest from the two biological replicates of the day 1 dosed tissue of *m/*z 646 (**a**) and 662 (**b**) in positive mode ionisation, and *m/z* 865 (C) in negative mode ionisation. The images show the distribution of the major fragment ions of amiodarone (**a**) and N-desyl amiodarone (**b**), and the parent ion 865 has two unique distributions with fragments for BMP(22:6) (*m/z* 283 and 327) and another ion, possibly PG 22:6; however, the MS/MS inclusion window is > 1 Da so other lipids may be included into this window. Full image area for **a** and **b** is 12.5 × 9.3 mm, and **c** is 11.8 × 7.0 mm

DESI MSI was carried out using a Waters Xevo G2-XS Q-ToF instrument with a DESI ion source (both Waters, UK) with square pixels of either 50 μm for the imaging MS and MS/MS repeat analysis of the single dosed tissues or 100 μm for the imaging of all dosing conditions in positive and negative ion modes. A scan time of 0.48 s was used in both cases, equating to stage speeds of 100 and 200 μm/s respectively. The mass range was set to 100–1200 for all MS analysis, and for MS/MS, the mass range was set from 50 to the target mass plus 5. All imaging was carried out using a capillary voltage of 4.5 keV for positive mode and 4.25 keV for negative mode, with a sampling cone set to 50 a.u. and a source block temperature of 100 °C. All mass spectrometry was carried out in sensitivity mode, and MS/MS was performed using collision-induced dissociation with a collision energy of 35 a.u. and a parent ion resolving quadrupole set to 4.7 a.u. The solvent system used in all cases was 95:5 methanol:water (v:v) with 0.001 mg/mL raffinose added for use as lock mass compound and internal standard. Prior to image acquisition, the detected ion intensity from rhodamine [M+H]^+^ at *m*/*z* 443.23 (from red Sharpie marker pen) for positive ion mode or *m/z* 666 (unknown ion from black Staedtler marker pen) for negative ion mode is checked with a quality control threshold of > 10^6^ required to carry out the experiment without further optimisation. Additionally, the characteristics of the eroded spot from the DESI spray were assessed on a thin film of rhodamine sublimated onto glass [21]: A tightly focussed, approximately circular region clearly eroding through the entire layer in ≤ 1 s is desirable. Different tissue sections were used for the data in Figs. 1 and 2 a and c. The repeat analysis was carried out in the following order; for the positive and negative ion mode comparison, positive ion mode was carried out first. For the MS and MS/MS imaging, MS imaging was carried out first, followed by MS/MS, and in the case of amiodarone and N-desyl amiodarone, amiodarone MS/MS was carried out prior to N-desyl amiodarone.

### Data processing and analysis

Data were converted from proprietary Waters .raw format into imzML using ProteoWizard [24] and the imzML converter [25], and imported into MATLAB (version 2017a and statistics and image processing toolbox, The MathWorks, Inc., Natick, MA, USA) using SpectralAnalysis [26]. Ion images were generated by integrating intensities across the *m/z* widths specified.

All data were registered using the MATLAB image processing toolbox (version 2017a image processing toolbox, The MathWorks, Inc., Natick, MA, USA) according to the schematic in Fig. S1 (see Electronic Supplementary Material, ESM). Firstly, binary masks of the tissue were created by performing *k*-means clustering on the data (*k* = 2, cosine distance, 3 replicates) [27]. Then, registration points were manually selected using the MATLAB function “cpselect”. A rigid transformation was the generated from these selection points using the positive mode data as the “fixed points”, and negative mode or MS/MS data as the “moving points”. This transformation was then applied to the full dataset and overlays of the newly registered ion images were created.

## Results and discussion

One of the key advantages of MSI over quantitative whole-body radiography and LC-MS approaches is the ability to spatially localise parent drug molecules and its constituent metabolites. This has been shown to be particularly important in drug toxicology studies where the drug and metabolites can have different toxicological effects and may localise to different tissue areas [28]. Analysing lung tissue by DESI in positive mode shows ions corresponding to the amiodarone [M+H]^+^ (*m/z* 646.04), along with metabolites previously identified by LC-MS but not by MSI (N-desyl amiodarone, and metabolites M8 and M11) [9] which have different spatial distributions (Fig. 1). Amiodarone appears to be highly localised which may be primarily near major airways as determined by a high-resolution optical image (ESM Fig. S2) and stained H&E image (ESM Fig. S3), while the metabolites are much more homogeneously distributed throughout the tissue. This can also be seen by analysing the univariate distribution of intensities in boxplots of the intensity in each of the four images. From these, the amiodarone outliers have much longer tails (ESM Fig. S4), reflecting the high intensity shown in low pixel numbers for these regions.

However, in MS analysis, even at high mass resolving power, a number of molecules are detected at the same measured *m/z* and if a mass error is introduced, this number increased even further. Therefore, to add confidence to any given molecular identity, fragmentation and analysis by tandem MS are required. This has been done previously by bulk measurement using LC-MS/MS [29], although this approach loses any spatial information, important in cases where multiple isomeric species are present in different spatial distributions. This problem is particularly relevant to drug metabolism studies since many metabolites have the same mass, but may differ in their toxicological effects [9]. To confirm the identity of a molecule and measure the spatial distribution, MS/MS imaging can be performed, and this has been demonstrated for a variety of different drug molecules [30, 31], although additional serial tissue sections would normally be required. Multiple product ion images from positively charge ions from the same section have been obtained using multiple reaction monitoring by Prideaux et al. [32]. To date, there are no reports of full scan and product ion data from multiple classes of analyte in positive and negative ion modes from a single section.

Since lung tissue is particularly fragile, collecting exact serial sections and alignment of these are exceptionally challenging. Fixation and embedding following inflation have been shown to improve tissue handling [33] for lipid analysis by MSI; however, this workflow is likely to delocalise drug and metabolite molecules and therefore is not suitable for spatial distribution studies and more fragile fresh frozen tissue must be used.

A major advantage of repeat analysis of the same section multiple times lies in the reduced complexity of registration routines required for data acquisition. For example, when data is acquired from serial sections of stable tissues, registration routines must be devised to account for deformation and warping of the sample during sectioning [34, 35]. In contrast, repeat analysis of the same section multiple times means there is no deformation of the sample and simple rigid registration routines can be applied to these data. In this study, we used the MATLAB “cpselect” function to manually define matching points between data acquired in positive and negative modes and used these as a basis for rigid transformation using the MATLAB functions “cp2tform” and “imtransform”. Other methods such as by normalised cross correlation could be used; however, the intention was to not introduce any bias into any further correlation-based analysis by the registration process [36].

**Fig. 3:**
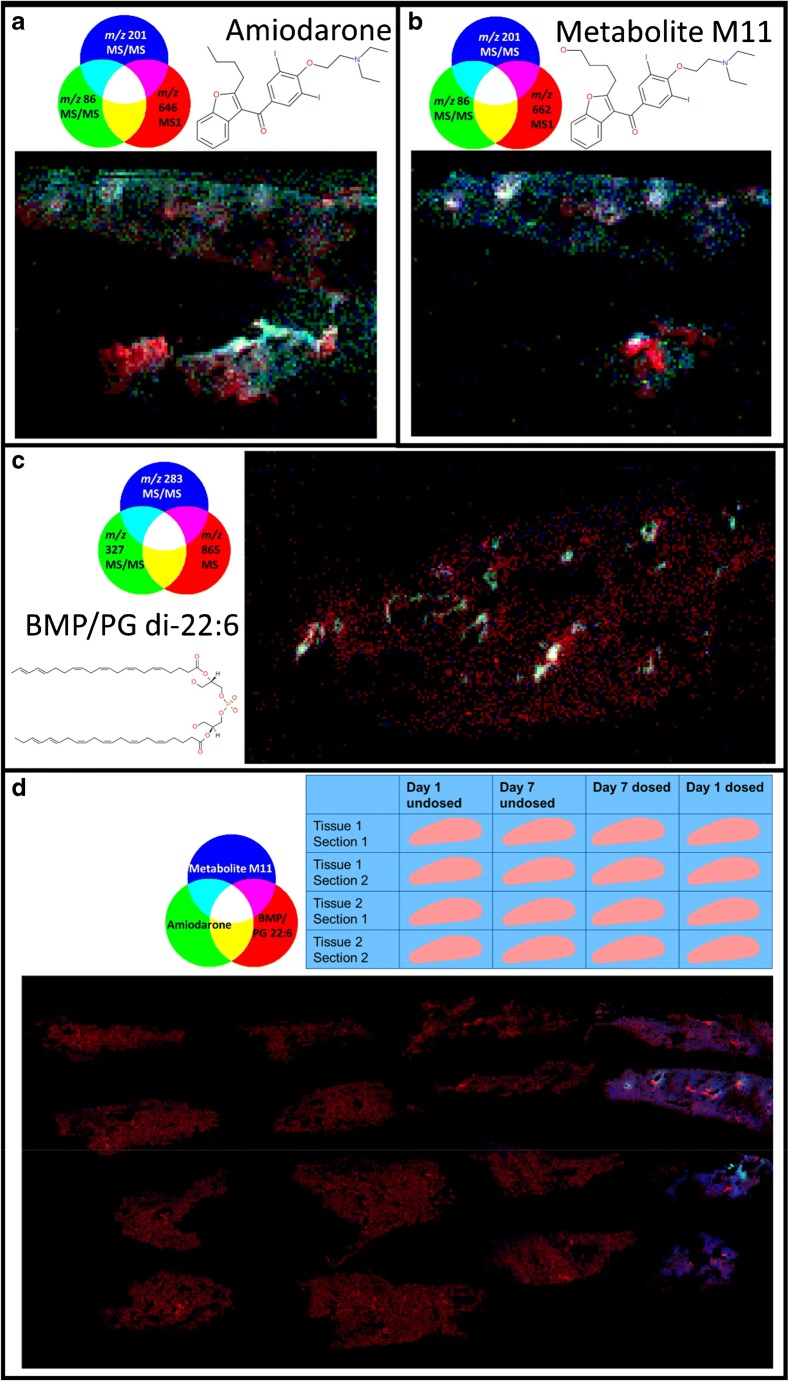
Registered RGB overlay images from repeat analysis experiments including the two biological replicates of the day 1 dosed tissue showing parent molecule MS1 (red) and major MS/MS fragments (blue and green) of amiodarone (**a**), metabolite M11 (**b**), and di-22:6-BMP or PG di-22:6 (**c**). Combining positive mode MS1 images of amiodarone and metabolite M11 and negative mode images of BMP di-22:6/PG di-22:6 shows the spatially localised increase in this lipid alongside the presence of drug and metabolite in the day 1 dosed tissue (**d**). Full image area for **a** and **b** is 12.5 × 9.3 mm, **c** is 11.8 × 7.0 mm and **d** is 46.5 × 23.3 mm

The approach was demonstrated by analysing two replicate lung tissue slices taken from a single lung 24 h following aerosol dosing with 6.25 mg/kg amiodarone. Repeat MS/MS analysis of the amiodarone [M+H]^+^ ion (*m/z* 646) resulted in known amiodarone fragment peaks (*m/z* 86.1, 100.1, 201.1 and 572.9) [37, 38] observed with similar apparent spatial distribution to the parent MS1 images (Fig. 2a). The overlapping spatial distribution is even more apparent when these images are registered and overlaid with one another (Fig. 3a). The spatial distribution of amiodarone metabolite ions was also studied using the same approach. The amiodarone metabolite M11 (*m*/*z* 662) exists in five possible isomeric forms (M11-1 to M11-5) with different possible oxidation locations [9]. The characteristic fragment peaks *m/z* 201, 116, 100 and 86 were observed, suggesting that the M11-3 and M11-4 molecules are present in these tissues (Figs. 2b and 3b). It is worth noting that the other forms may also still be present in concentrations that are below the limit of detection. In this example, these ions had similar spatial distributions to one another, indicating that they are found within the same tissue regions, but these methodologies could also be used to analyse spatial differences in isomeric metabolites which are not co-localised. Thus, the use of repeat MS/MS analysis in this manner allows an initial untargeted evaluation of the identity and spatial location of drugs and their metabolites in a given tissue, which is a key step forward in drug discovered and toxicology studies. In addition to being able to map the spatial location of drug and its metabolites, MSI is also capable of analysing drug-induced changes to tissues by determining the identity and measuring the spatial distribution of selected endogenous molecules. Peak-picked mean spectra from both positive and negative mode analyses were matched to the HMDB database using previously described methods [39]. Briefly, peaks were filtered using a ppm mass error of < 5 and a C_12_–C_13_ isotope image correlation of > 0.7. A large number of endogenous molecules of interest were tentatively identified as summarised by Tables S1 and S2 (see ESM).

**Fig. 4:**
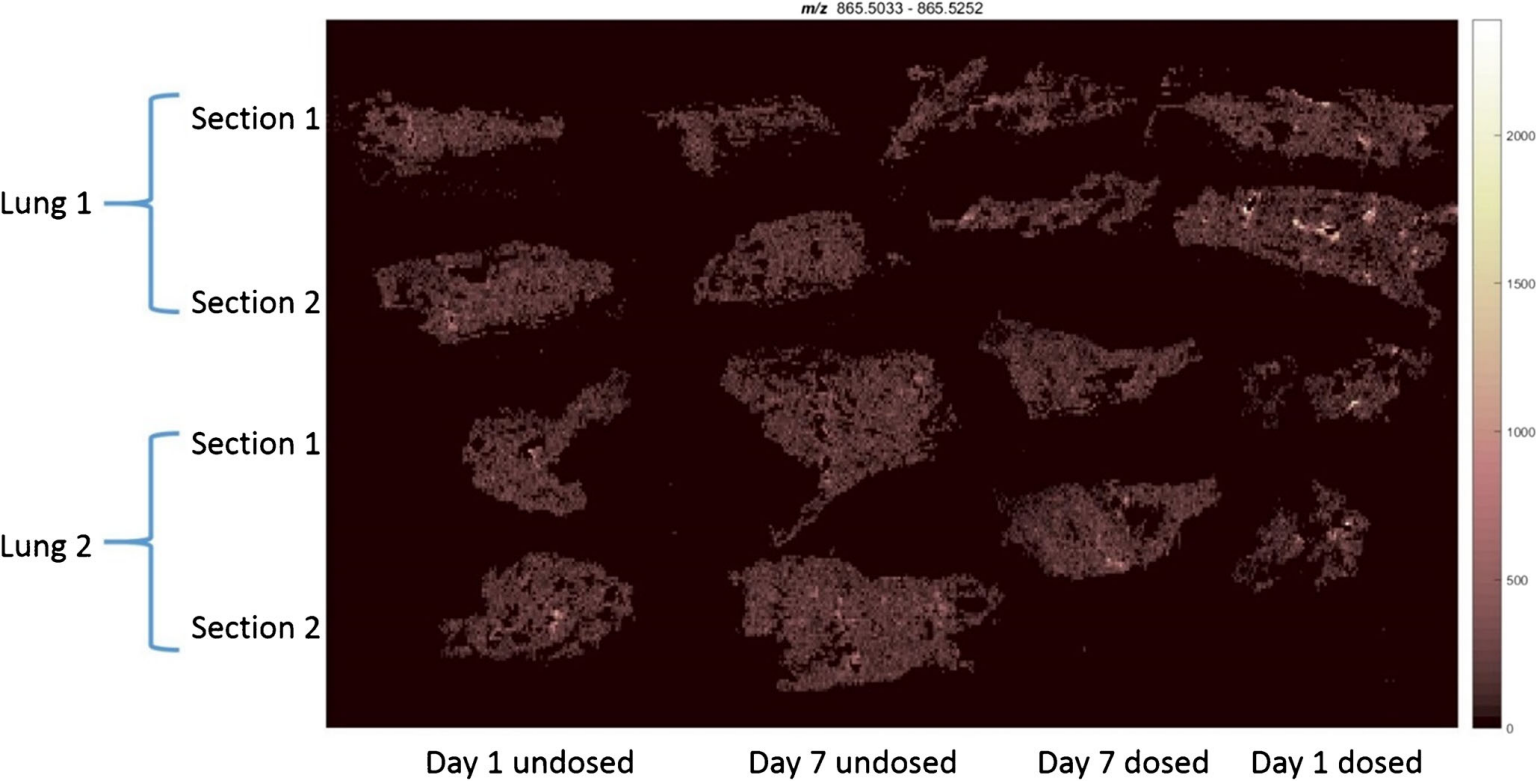
Ion image of *m/z* 865.51 showing a higher distribution of in a highly localised area of the tissue (possibly major airways) along with a background in the tissue. This could be the two isomeric species di-22:6-BMP in the airways and di-22:6 PG throughout the tissue. Full image area for these data is 46.5 × 23.3 mm

Since orally administered amiodarone is known to induce lipidosis in several organs, including the lung, it was hypothesised that di-22:6-BMP [40] would be detectable in amiodarone-treated lung tissues [41]. Using MSI analysis, a mass corresponding to the molecule di-22:6-BMP (detected nominal mass 865.51: ppm error 8.6554) was observed in lung slices taken from animals sacrificed 24 h following aerosol exposure. The signals were localised to areas surrounding major airways and appeared similar in spatial distribution to amiodarone (Fig. 4). The di-22:6-BMP signal appeared to return to endogenous levels by day 7 following the final amiodarone exposure, indicating a resolution to the mild form of drug-induced lipidosis. Interestingly, conventional histopathology of the lung tissue at all time points following aerosol dosing with amiodarone did not show lipidosis development (data not shown), as characterised by visual observation of an increased number of foamy macrophages containing accumulated lipids. The lack of pronounced lipidosis in the aerosol dosing model was attributed to a lower concentration of amiodarone accumulating in the lung compared with published oral dosing studies (300 mg/kg) [42]. This discrepancy clearly demonstrates that modern MS techniques, particularly in this case with the use of MSI, have pronounced advantages over conventional histopathology in terms of sensitivity and the ability to discern the spatial distribution of molecular species of interest.

Amiodarone and its metabolites are therefore detected in positive mode as [M+H]^+^ ions, while di-22:6-BMP is more easily is commonly detected in negative ion mode as [M−H]^−^ ions. In order to analyse both these molecules in a single tissue, serial sections could be taken, but not without the tissues previously discussed.

di-22:6-BMP is also an isomer of any PG 44:12 lipid species, with many possible lipid side chain combinations. Therefore, to confirm the exact identity of these lipid species, it is necessary to fragment these ions and perform tandem MS experiments. Many of the fragment ions of di-22:6-BMP and di-22:6 PG are common, since they have the same lipid side chains; therefore, characterisation would normally require an orthogonal means of separation [12]. However, by measuring the spatial distributions of fragments previously reported in literature to belong specifically to di-22:6-BMP, *m/z* 283.2 and 327.2 [40], it was observed that these ions were localised to the same regions, possibly surrounding major airways. These ions match molecular formulae of C_21_H_31_^−^ (for *m/z* 283.2) and C_22_H_31_O_2_^−^ (for *m/z* 327.2) which could belong to the 22:6 lipid side chains. Although these side chains should also be present for PG lipids, these fragment ions had a very different spatial distribution compared with the parent *m/z* 865.5 ion, and since amiodarone is a compound known to induce lipidosis, and these fragments have previously been reported for di-22:6-BMP, it is proposed that the fragments shown correspond to di-22:6-BMP and that spatially resolving MS/MS fragments can be used to differentiate and confirm a compounds’ identity.

As stated previously, a major advantage of the repeat analysis technique is the ability to overlay the images from positive mode analysis of amiodarone (*m/z* 646 positive mode), metabolite M11 (*m/z* 662 positive mode) and di-22:6-BMP (*m/z* 865 negative mode) to show how their spatial distributions are related to one another (Fig. 3d). These molecules have then all been confirmed by MS/MS imaging to provide confidence in their assignment and to visualise their distributions in the same section. Furthermore, the amiodarone metabolite M11 is known to be forms M11-3 and M11-4 based on the presence of specific MS/MS fragment peaks.

## Conclusions

Repeat analysis of a single tissue section by DESI MSI using multiple modalities and polarities can provide a wealth of complimentary information. This can be used to measure the spatial distribution of drugs and exogenous and endogenous metabolites all from a single sample, and allows confirmation of molecular identities. Here we use this methodology to discover spatially distinct drug-induced lipidosis in lung tissues after inhaled amiodarone dosing, and regions of lipidosis correlate with regions of high drug and metabolite accumulation. This is of particular importance for precious or fragile samples such as lung, where either sample volume or stability precludes collection of additional serial sections. Currently the analyses of these data are minimal; however, using these methodologies, more sophisticated computational and statistical analyses, such as co-localisation analyses, and image fusion methods could be applied to mine the wealth of data provided my these multimodal repeat analysis studies. In further work, the study of whether repeat analysis may result in some analytes being delocalised or depleted to different degrees by the DESI sampling process will be of interest. We hypothesise that these will be dependent on the physicochemical properties of the analytes, DESI parameters selected and solvent composition.

## Electronic supplementary material

ESM 1 (PDF 1052 kb)
