# Supplementary material for: Imaging drugs, metabolites and biomarkers in rodent lung: a DESI MS strategy for the evaluation of drug-induced lipidosis
Source: Anal Bioanal Chem. 2019 Nov 27;411(30):8023–32. doi: 10.1007/s00216-019-02151-z (PMC6920235; doi:10.1007/s00216-019-02151-z)
Supplement: Supplementary file 1 — (PDF 1052 kb) [file 216_2019_2151_MOESM1_ESM.pdf]

Analytical and Bioanalytical Chemistry

Electronic Supplementary Material

Imaging drugs, metabolites, and biomarkers in rodent lung: A DESI MS strategy for the evaluation of drug-induced lipidosis

Alex Dexter, Rory T. Steven, Aateka Patel, Lea Ann Dailey, Adam J. Taylor, Doug Ball, Jan Klapwijk, Ben Forbes, Clive P. Page, Josephine Bunch

Contents

Figure S1 Schematic of the registration workflow to combine and overlay either positive and negative mode or MS and MS/MS data

Figure S2 High resolution optical image from the lung tissues to highlight the locations of amiodarone accumulation

Figure S3 High resolution h&e stained lung tissue for the amiodarone dosed samples post DESI analysis

Figure S4 Boxplot of the intensities of amiodarone and major metabolites

Table S1 List of tentative molecular assignments grouped according to molecular class from the positive mode DESI data

Table S2 List of tentative molecular assignments grouped according to molecular class from the negative mode DESI data

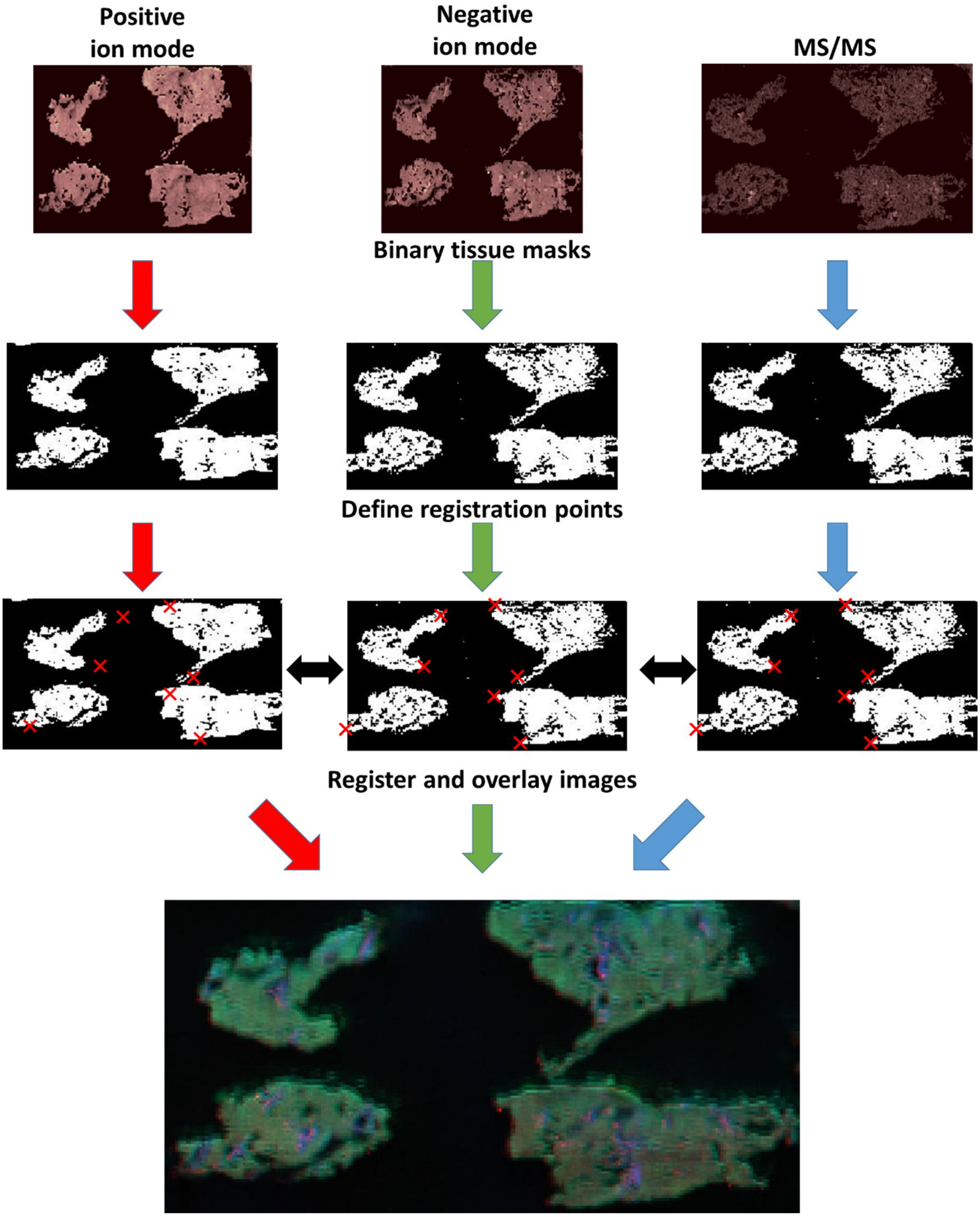

Fig. S1 Schematic of the registration workflow to combine and overlay either positive and negative mode or MS and MS/MS data

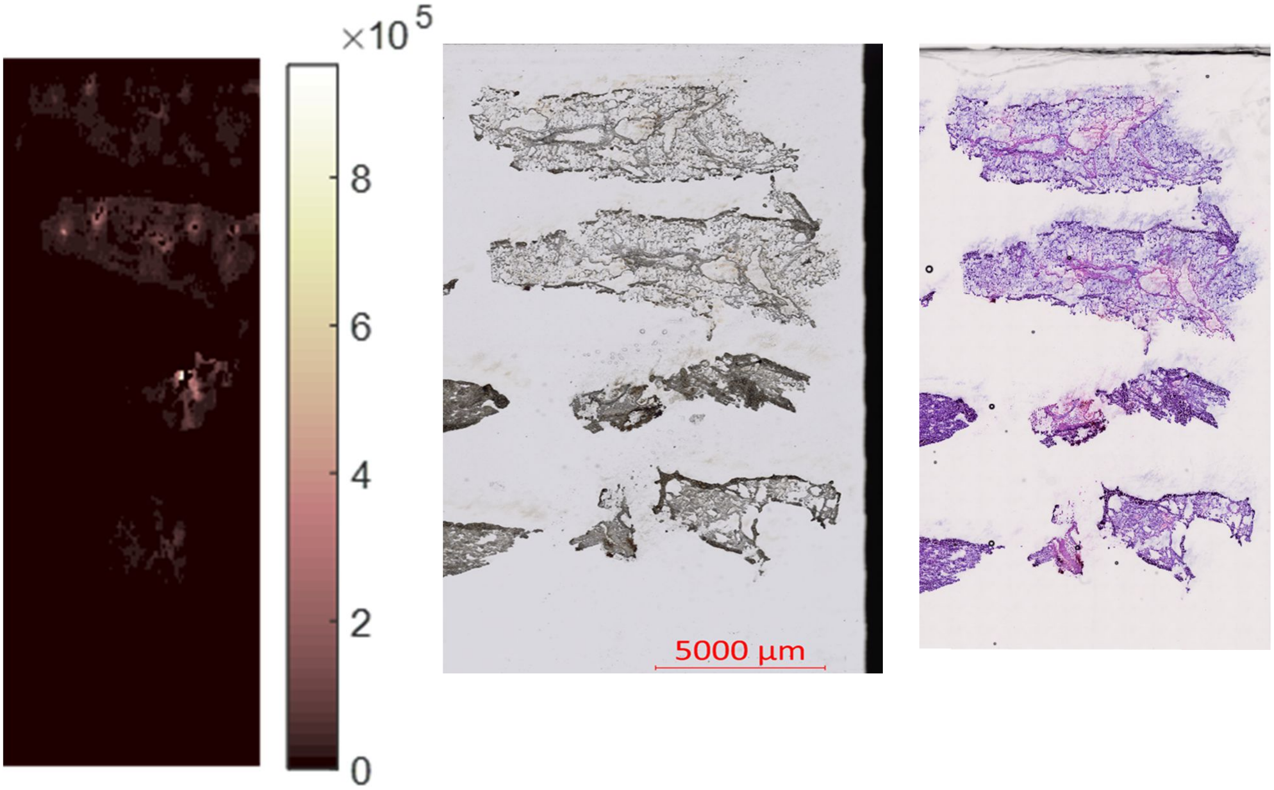

Fig. S2 High resolution optical image from the lung tissues to highlight the locations of amiodarone accumulation

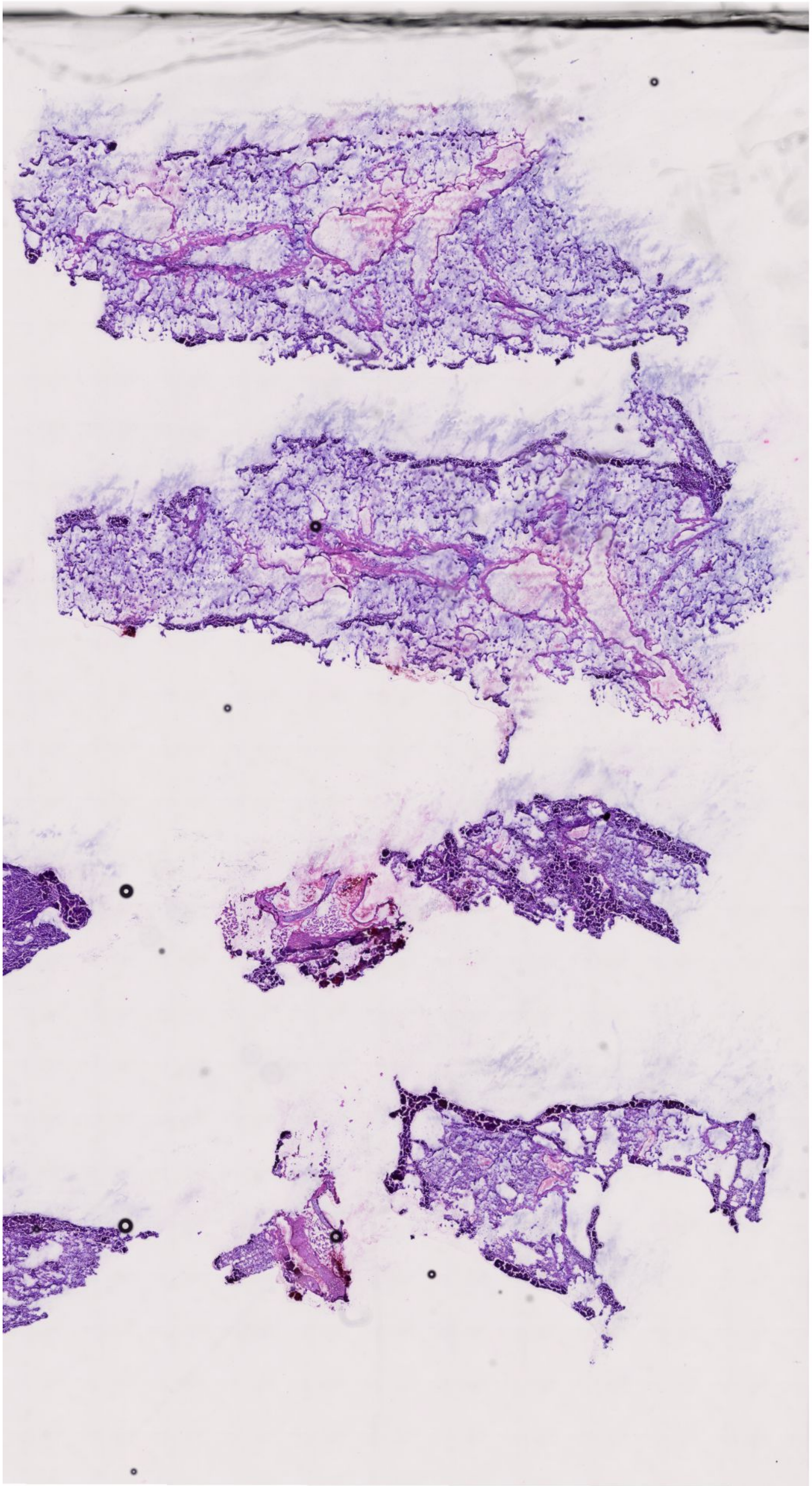

Fig. S3 High resolution h&e stained lung tissue for the amiodarone dosed samples post DESI analysis

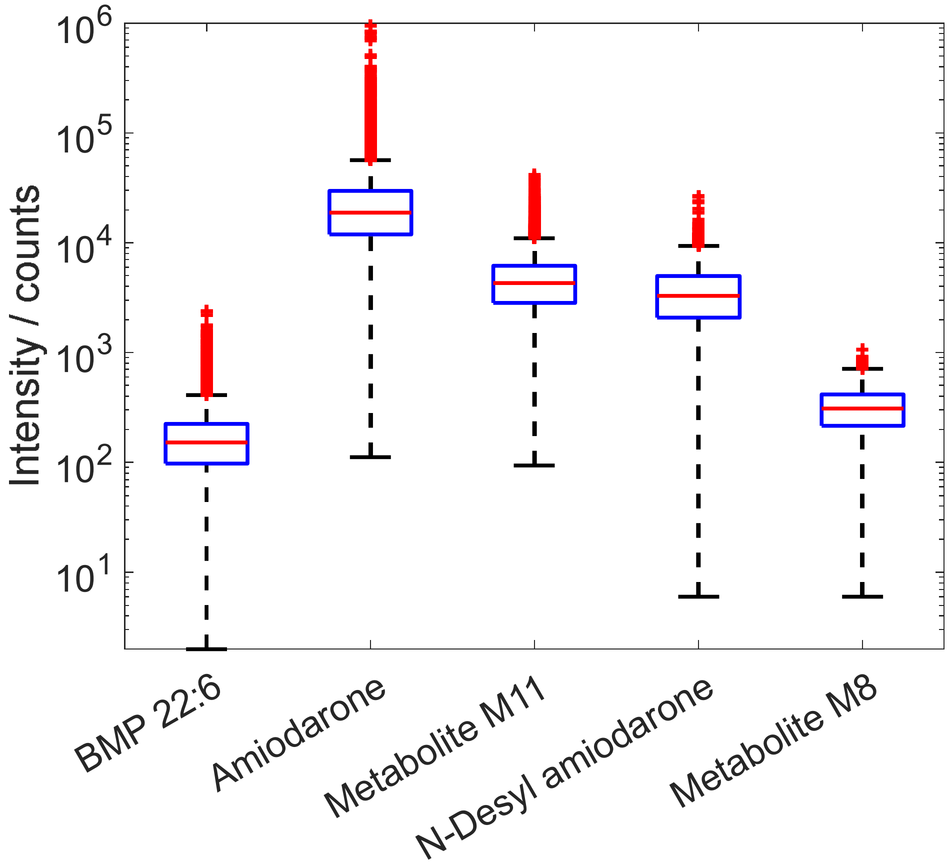

Fig. S4 Boxplot of the intensities of amiodarone and major metabolites

Table S1 List of tentative molecular assignments grouped according to molecular class from the positive mode DESI data

Molecule class	Frequency
TG lipid	67
PS lipid	11
PI lipid	6
PG lipid	18
PE lipid	20
PC lipid	35
PA lipid	22
DG lipid	22
CE lipid	9
Other small metabolites	880

Table S2 List of tentative molecular assignments grouped according to molecular class from the negative mode DESI data

Molecule class	Frequency
TG lipid	10
PS lipid	9
PI lipid	8
PG lipid	12
PE lipid	17
PC lipid	8
PA lipid	8
DG lipid	17
CE lipid	2
MG lipid	3
Lipid side chains	5
Other small metabolites	279
